# Integrative analysis of lactylation related genes in prostate cancer: unveiling heterogeneity through single-cell RNA-seq, bulk RNA-seq and machine learning

**DOI:** 10.3389/fphar.2025.1634985

**Published:** 2025-08-04

**Authors:** Chenghao Zhou, Lifeng Ding, Huailan Wang, Gonghui Li, Lei Gao

**Affiliations:** Department of Urology, Sir Run Run Shaw Hospital, Zhejiang University School of Medicine, Hangzhou, China

**Keywords:** prostate cancer, lactylation, prognostic biomarker, machine learning, personalized treatment, immune microenvironment

## Abstract

**Introduction:**

Lactylation, a post-translational modification characterized by the attachment of lactate to protein lysine residues on proteins, plays a pivotal role in cancer progression and immune evasion. However, its implications in immunity regulation and prostate cancer prognosis remains poorly understood. This study aims to systematically examine the impact of lactylation-related genes (LRGs) on prostate cancer.

**Methods:**

Single-cell and bulk RNA sequencing data from patients with prostate cancer were analyzed. Data were sourced from TCGA-PRAD, GSE116918, and GSE54460, with batch effects mitigated using the ComBat method. LRGs were identified from exisiting literature, and unsupervised clustering was applied to assess their prognostic siginificance. The tumor microenvironment and functional enrichment of relevant pathways were also evaluated. A prognostic model was developed using integrative machine learning techniques, with drug sensitivy analysis included. The mRNA expression profiles of the top ten genes were validated in clinical samples.

**Results:**

Single-cell RNA sequencing revealed distinct lactylation signatures across various cell types. Bulk RNA-seq analysis identified 56 prognostic LRGs, classifying patients into two distinct clusters with divergent prognoses. The high-risk cluster exhibited reduced immune cell infiltration and increased resistance to specific targeted therapies. A machine learning-based prognostic signature was developed, demonstrating robust predictive accuracy for treatment responses and disease outcomes.

**Conclusion:**

This study offers a comprehensive analysis of lactylation in prostate cancer, identifying potential prognostic biomarkers. The proposed prognostic signature provides a novel approach to personalized treatment strategies, deepening our understanding of the molecular mechanisms driving prostate cancer and offering a tool for predicting therapeutic responses and clinical outcomes.

## Background

Prostate cancer (PCa) is a leading malignancy in men, is shaped by a complex interplay of genetic, metabolic, and immunological factors that drive its progression and therapeutic resistance (Chen et al., 2021). The disease’s heterogeneity is reflected in its diverse genetic landscape, with notable genomic alterations, including TMPRSS2-ERG fusions and SPOP mutations, emerging in the early stages (He et al., 2022). Next-generation sequencing has revolutionized our understanding of PCa’s genomic landscape, uncovering a range of genetic abnormalities that are linked to disease progression and resistance to treatment (Fujita and Nonomura, 2019; Guo et al., 2024). Recent studies highlight the importance of metabolic shifts within the tumor microenvironment (TME), particularly the roles of lactic acid and lactylation in promoting cancer advancement and immune evasion (Zha et al., 2024).

Lactylation, the covalent attachment of lactate to protein lysine residues, is gaining recognition as a critical post-translational modification (PTM) that bridges metabolism and epigenetics, with profound implications for cancer biology (Brown and Ganapathy, 2020; Lv et al., 2023). In PCa cells, metabolic reprogramming in prostate cancer cells, characterized by the Warburg effect, leads to a shift towards aerobic glycolysis, resulting in lactate production even in the presence of oxygen (Luo et al., 2022). While previously considered a metabolic anomaly; lactate is now understood to function as a signaling molecule and a regulator of gene expression through lactylation. Once regarded solely as a byproduct of anaerobic metabolism, lactate is now recognized for its roles in systemic metabolism, cellular signaling, and as a substrate for oxidative metabolism in other tissues (Li et al., 2022). In PCa, lactate metabolism and lactylation contribute to immune evasion, angiogenesis, and the modulation of tumor microenvironment (TME).

The TME in PCa is characterized by hypoxia and acidosis, conditions that foster promote lactate accumulation (Liang et al., 2024). Through its receptor GPR81, lactate exerts a significant impact on cellular metabolism and tumor growth, independent of monocarboxylate transporters, protons, and glucose metabolism (He et al., 2024). Lactate also stabilizes hypoxia-inducible factor-1α (HIF-1α), a key regulator of the hypoxic response, which in turn activates the transcription of genes involved in tumorigenesis (Berglund et al., 2018). Additionally, lactate influences the immunological landscape of PCa by modulating immune cell function, facilitating immunosuppression and immune evasion. Lactate accumulation in the TME acidifies the environment, impairing T lymphocyte activity and inducing tumor-associated macrophages (TAMs) to adopt a pro-tumorigenic M2 phenotype (Luo et al., 2022).

This study provides a comprehensive analysis of lactylation-associated data derived from single-cell and bulk RNA sequencing, utilizing multiple databases to explore gene expression patterns. A novel prognostic biomarker was developed based on lactylation-related genes (LRGs). This biomarker evaluated the relationship between the LRG signature and various clinical and pathological features, as well as its correlation with PCa progression. Additionally, this study investigated the signature’s association with the TME, genetic mutations, and the effectiveness of immuno- and chemotherapy with PCa. Lastly, the mRNA expression profiles of top ten genes were valdated using ten paired prostate cancer clinical samples.

## Materials and methods

### Data collection and processing

RNA-seq data and corresponding clinical information for TCGA-PRAD were obtained from UCSC-XENA (https://xenabrowser.net/datapages/). Gene microarray data and clinical details from 248 patients with PRAD in the GSE116918 cohort and 106 patients in the GSE54460 cohort were retrieved from the Gene Expression Omnibus (GEO) database. The ComBat method from the sva package was applied to integrate and adjust for batch effects across the GSE116918, GSE54460, and TCGA-PRAD datasets. Public cancer databases, including GSCA (Liu et al., 2022), Tumor Immune Dysfunction and Exclusion (TIDE) (Jiang et al., 2018), and TISCH2 (Han et al., 2022), were also utilized in the study. As the datasets were publicly available, approval from an Ethical Review Committee and informed consent were not necessary. Patients without prognostic information or expression profiles were excluded from the analysis. The single-cell sequencing dataset of GSE176031 was downloaded from TISCH2, which included 19,969 genes and 15,339 cells. The filtered dataset was further analyzed using the Seurat package, with PCA and t-SNE applied for effective cell sample clustering. The COSG package was utilized for detailed cell type annotation and key gene selection in single-cell data (Dai et al., 2022). To identify genes linked to lactylation-related genes (LRGs), 327 genes were compiled from previously published studies (PMID37242427, PMID35761067. Supplementary Table S1).

We also employed the Single-Cell Identification of Subpopulations by Correlating with bulk Sample phenotypes (SCISSOR) method to investigate, at single-cell resolution, how LRGS relates to prognostic phenotypes, by jointly analyzing survival outcomes and transcriptomic data from the combined cohort (Sun et al., 2022).

### Unsupervised clustering of lactylation-related genes

For unsupervised clustering of the LRGs, we utilized the “ConsensusClusterPlus” R package (Wilkerson and Hayes, 2010). Agglomerative clustering was conducted using a spearman correlation distance metric was performed, with 80% of the samples resampled for 10 repetitions. The optimal number of clusters was determined using an empirical cumulative distribution function plot. Kaplan-Meier analysis was performed to assess the RFS (Recurrence Free Survival) of patients with PRAD across different clusters.

### Evaluation of the cell tumor microenvironment and functional enrichment of pathways

An immune landscape specific to patients with PRAD was developed to explore the regulatory influence of the LRG score model on the TME. The immune gene signature encompasses the expression of key immune checkpoints and the infiltration characteristics of diverse immune cells. Gene signatures of immune cells were sourced from seven different platforms using the IOBR package: TIMER, CIBERSORT, CIBERSORT-ABS, QUANTISEQ, MCPCOUNTER, XCELL, and EPIC (Zeng et al., 2024). GO and KEGG pathway analyses were conducted using the clusterProfiler package (v4.6.2) (Wu et al., 2021). Further, gene set variation analysis (GSVA) and gene set enrichment analysis (GSEA) were conducted using the GSVA package (v1.46.0) to assess the various gene signatures (Hänzelmann et al., 2013).

### Development of a prognostic model using integrated machine learning techniques

To ensure a reliable identification of LRGs, 10 machine-learning algorithms were integrated to enhance accuracy and stability. These algorithms included various techniques such as random survival forest (RSF), elastic network (Enet), Lasso, Ridge, stepwise Cox, CoxBoost, partial least squares regression for Cox (plsRcox), supervised principal components (SuperPC), generalized boosted regression modeling (GBM), and survival support vector machine (survival-SVM). The signature generation process involved: (a)applying these algorithm combinations to the 56 identified prognostic LRGs to build predictive models in the combined cohort, and (b) cross-validating all models using separate datasets (GSE116918, GSE54460, and TCGA-PRAD). The Harrell’s concordance index (C-index) was computed for each model across all validation datasets, and the model with the highest average C-index was deemed as optimal.

### Potential drug sensitivity analysis

The oncoPredict R package (Maeser et al., 2021) was used to predict the chemosensitivity of patients with PRAD based on their LRG Risk Score (LRGRS). This approach correlates patients’ tissue gene expression profiles with those of cancer cell lines to estimate the half-maximal inhibitory concentration (IC50). The Wilcoxon test was employed to compare differences in drug IC50 value between high- and low-risk groups, with statistical significance set at p < 0.05.

### Collection of patient samples, RNA extraction, and quantitative real-time PCR

The study was approved by the Ethics Committee of Sir Run Shaw Hospital, Zhejiang University, with all patients providing written informed consent. All procedures were performed in accordance with the Declaration of Helsinki. Expression of LRGs was assessed in 20 tissue samples collected from randomly selected patients. The specimen collection process involved the following steps: patient selection and consent, tissue collection, fixation, embedding, sectioning, histopathological analysis, sample storage, and RNA extraction. Tissue RNA was extracted using TRIzol reagent (Invitrogen, CA, United States). First-strand cDNA synthesis was performed using the HiFiScript cDNA Synthesis Kit (CWBio), and real-time quantitative PCR (RT-qPCR) was conducted using the SYBR Green method on a Roche LightCycler^®^ 480 System. Primer sequences used in this study are listed in Table 1.

**TABLE 1:** Primers of top ten LRGs.

Gene symbol	Primer F	Primer R
RBM17	AGT​GGA​GAC​CAG​TGA​CTC​AAA	CTG​GGG​CGA​GGA​CTG​TAC​T
MTA1	ACG​CAA​CCC​TGT​CAG​TCT​G	GGG​CAG​GTC​CAC​CAT​TTC​C
PRAM1	GCA​GCC​TGA​GTT​GAG​TAC​CTT	GGC​ACG​GAC​TTC​TTA​GGG​AG
RACGAP1	ATG​ATG​CTG​AAT​GTG​CGG​AAT	CGC​CAA​CTG​GAT​AAA​TTG​GAC​TT
VIM	GAC​GCC​ATC​AAC​ACC​GAG​TT	CTT​TGT​CGT​TGG​TTA​GCT​GGT
MKI67	ACG​CCT​GGT​TAC​TAT​CAA​AAG​G	CAG​ACC​CAT​TTA​CTT​GTG​TTG​GA
MNDA	AAC​TGA​CAT​CGG​AAG​CAA​GAG	CCT​GAT​TCG​GAG​TAA​ACG​AAG​TG
CCNA2	CGC​TGG​CGG​TAC​TGA​AGT​C	GAG​GAA​CGG​TGA​CAT​GCT​CAT
RBM10	ATG​GAG​TAT​GAA​AGA​CGT​GGT​GG	TCC​CGG​TAG​TCG​TGG​TCT​C
KIF2C	CTG​TTT​CCC​GGT​CTC​GCT​ATC	AGA​AGC​TGT​AAG​AGT​TCT​GGG​T

### Statistical analysis

All statistical analyses were conducted using R software (version 4.4.1). A chi-squared test was used to compare clinical characteristics between the training and internal validation sets. The Wilcoxon test, a non-parametric method, was used to assess differences between variables that did not follow a normal distribution. Differentially expressed genes (DEGs) were evaluated for statistical significance using FDR-corrected p-values. Biochemical recurrence-free survival (BCR) among subgroups was compared using Kaplan-Meier survival analysis and the log-rank test with the “survival” package in R. Independent prognostic factors were analyzed using univariate and multivariate Cox regression models. Model performance was evaluated using ROC curve analysis and AUC calculation with the “timeROC” package in R. Spearman’s correlation analysis was conducted to assess the relationship between risk scores and immune cell infiltration. A Student’s t-test was used to analyze qRT-PCR results. Statistical significance was defined as p < 0.05, unless otherwise stated.

## Results

### Lactylation characteristic in single-cell transcriptome

**FIGURE 1:**
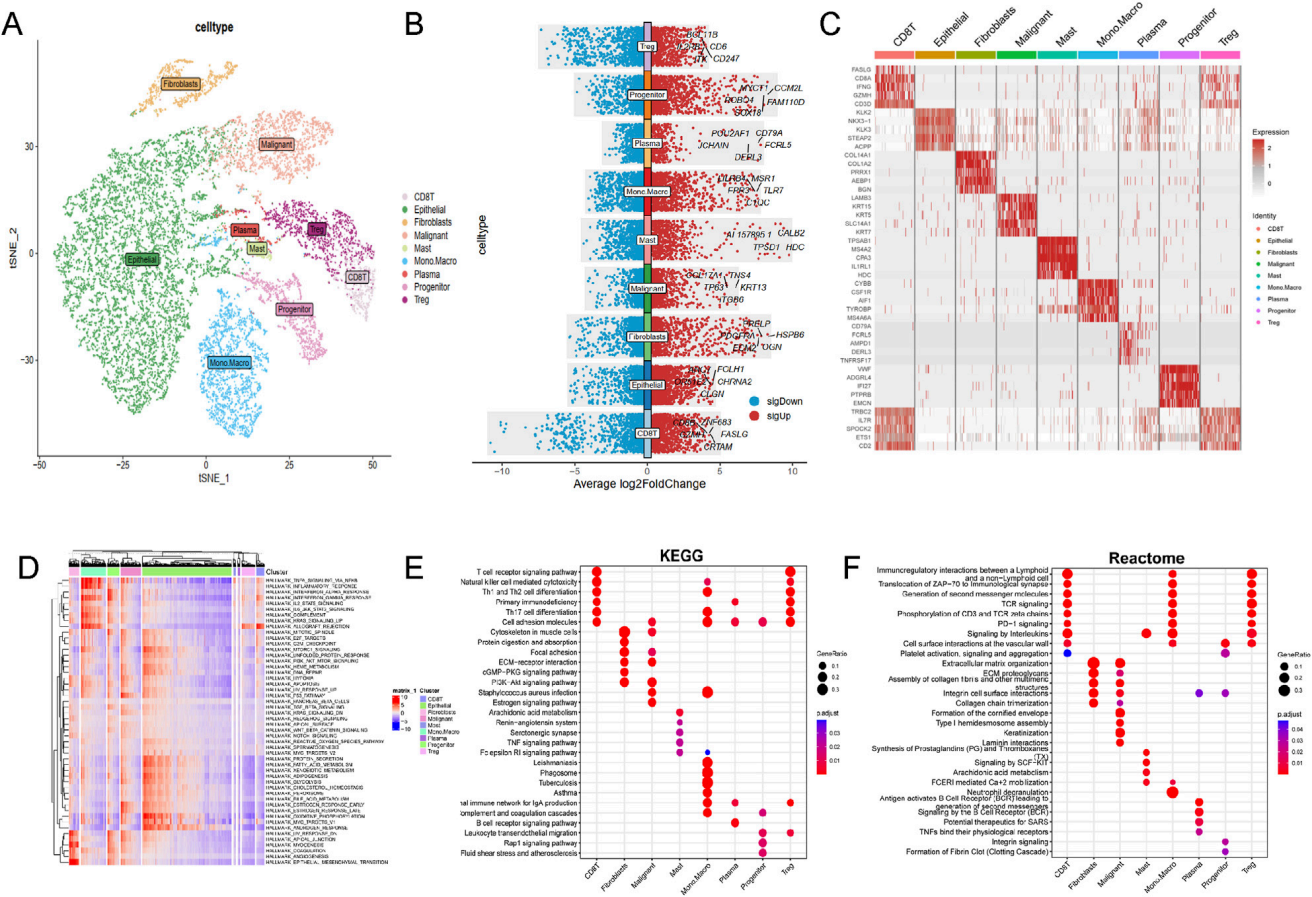
Single-cell RNA sequencing data analysis in PRAD cohort. **(A)** The results of the dimension reduction cluster analysis are shown in the t-SNE diagram. **(B)** Violin plots showing the distribution of average log2 fold change in gene expression for significant genes across different cell types. **(C)** Heatmap representation of gene expression profiles across various cell types. **(D)** Hallmark pathways, **(E)** KEGG pathways and **(F)** Reactome enrichment analysis among different cell types.

Single-cell RNA sequencing data from 34,155 PCa cellsusing were anlyzed using the TISCH2 dataset. Dimensionality reduction was performed on the top 2,000 variant genes via principal component analysis (PCA) and t-distributed stochastic neighbor embedding (t-SNE). Cells were clustered into 40 groups with a resolution of 0.8. Ten primary cell clusters were identified based on marker genes specific to various cell types: CD8T, epithelial, fibroblasts, malignant, mast, mono. macro, plasma, progenitor, and Treg cells (Figure 1A). Differential gene expression is illustrated in the volcano plot (Figure 1B), while the heatmap highlights the top five marker genes for each cell population (Figure 1C). Functional enrichment analysis of these cell types were analyzed based on Hallmark, KEGG and Reactome pathways (Figures 1D–F).

**FIGURE 2:**
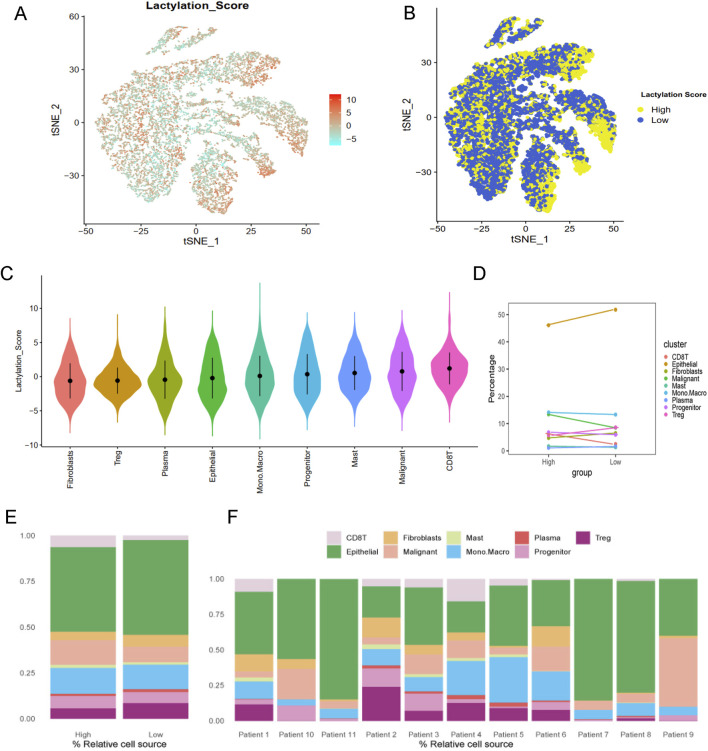
Lactylation characteristic in the single-cell transcriptome. **(A)** The activity score of lactylation in each cell types with t-SNE plot. **(B)** The high- and low-lactylation score group with t-SNE plot. **(C)** The distribution of the lactylation score in different cell types. **(D)** The dotplot and **(E)** barplot of different cell type percentage between high- and low-lactylation score. **(F)** The barplot of cell type percentage among different patients.

Lactylation activity was assessed using the “AddModuleScore” function from the Seurat package to evaluate the expression levels of a 257-gene set across various cell types (Figure 2A). Cells were categorized into high- and low-lactylation groups based on their lactylation activity (Figure 2B). Among the 9 cell types, CD8T and malignant cells exhibited significantly higher lactylation activity (Figures 2C–F).

### Identification of lactylation patterns in bulk RNA-seq

**FIGURE 3:**
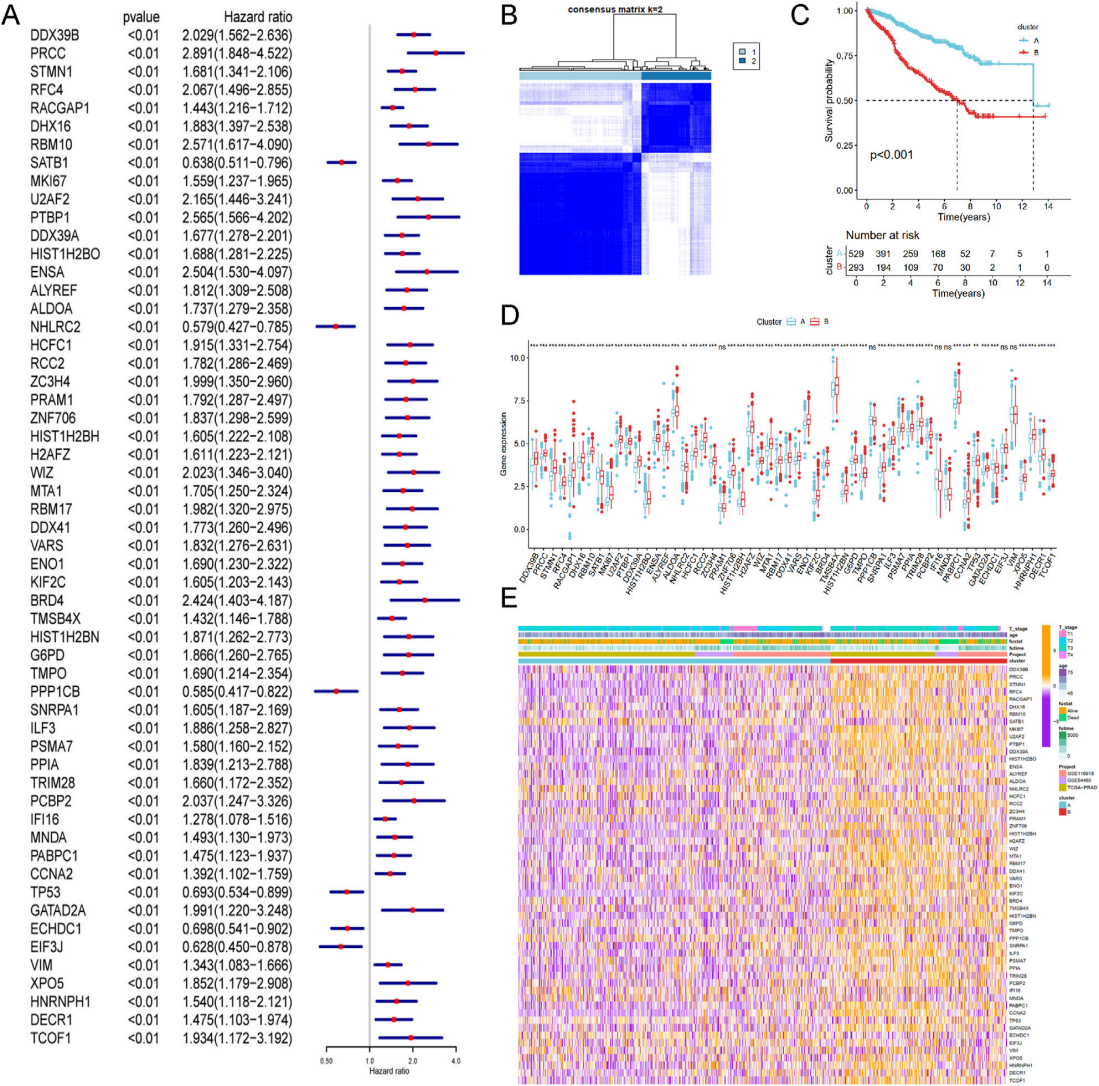
Identification of lactylation related molecular subtypes and comprehensive pathway enrichment analysis in combined cohorts. **(A)** Forest plot displaying the results of cox analysis shows 56 genes with prognostic value (Pvalue <0.01). **(B)** Consensus clustering matrixes was generated for values as k = 2. **(C)** Kaplan-Meier survival curves for these two distinct clusters (p < 0.001). **(D)** Gene set enrichment analysis (GSEA) plot showing the enrichment of gene sets in two clusters. **(E)** Heatmap of gene expression levels among different clinical characteristics (age, t stage, survival status).

To enhance statistical power and diversity, the GSE116918, GSE54460 and TCGA-PRAD datasets were integrated using the ComBat method. Resulting in a merged cohort consisting of 15,245 genes and 822 patients. Univariate Cox regression analysis was conducted to investigate the prognostic significance of 327 LRGs. Fifty-six genes were found to be significantly associated with RFS (P value <0.01, Figure 3A). Unsupervised cluster analysis based on the 56 prognostic genes categorized samples from the combined cohort into two distinct groups, Cluster A and Cluster B (Figure 3B). Survival analysis revealed that Cluster B was associated with a worse prognosis that Cluster A (P < 0.05, Figure 3C). Figure 3D presents a boxplot illustrating the variations in prognostic genes between Cluster A and Cluster B. The heatmap demonstrated the association of prognostic genes expressions among age, survival status and T stage in different clusters (Figure 3E), suggesting a worse clinical outcome.

### Differences in biological characteristics between lactylation subtypes

**FIGURE 4:**
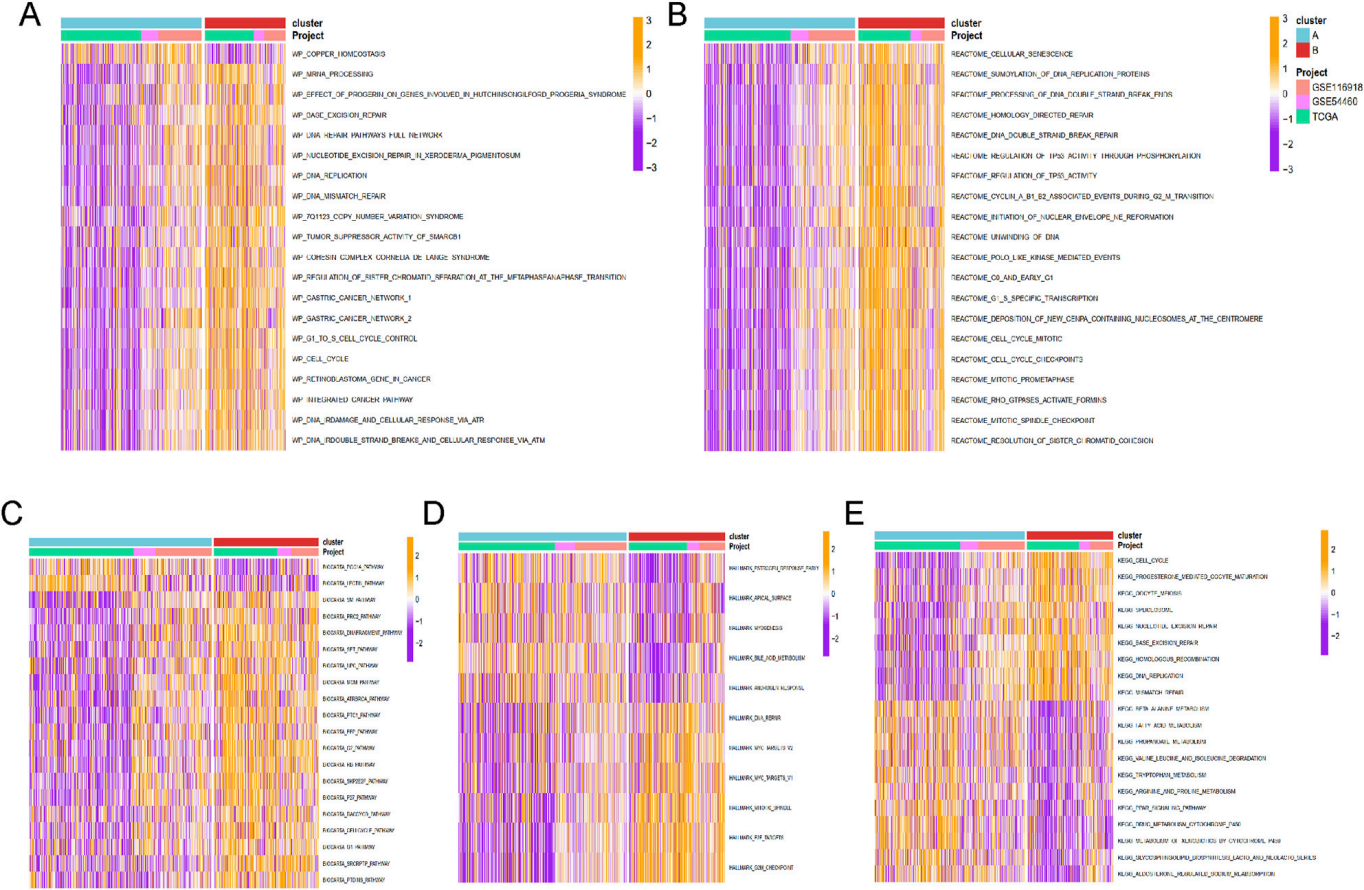
The functional enrichment analysis results of GSVA methods between different clusters. The **(A)** wikipathway, **(B)** Reactome, **(C)** Biocarta, **(D)** HALLMARK, and **(E)** KEGG pathways enrichment of cluster A and B groups.

Enrichment analysis for Cluster A and Cluster B was conducted using the GSVA method, with five types of analyses performed. Pathway enrichment analysis revealed that Cluster B was significantly enriched in cancer-related pathways, including base excision repair, DNA replication, DNA mismatch repair, cell cycle, and integrated cancer pathways (Figure 4A). These results were consistent with the findings from Reactome and Biocart enrichment analyses (Figures 4B–E). In contrast, Cluster A exhibited significant enrichment in hallmark pathways such as estrogen response early, apical surface, myogenesis, and androgen response (Figure 4D). KEGG pathway analysis also indicated that Cluster A was enriched in several metabolic pathways, including beta-alanine metabolism, fatty acid metabolism, propanoate metabolism, tryptophan metabolism, and drug metabolism via cytochrome P450 (Figure 4E).

**FIGURE 5:**
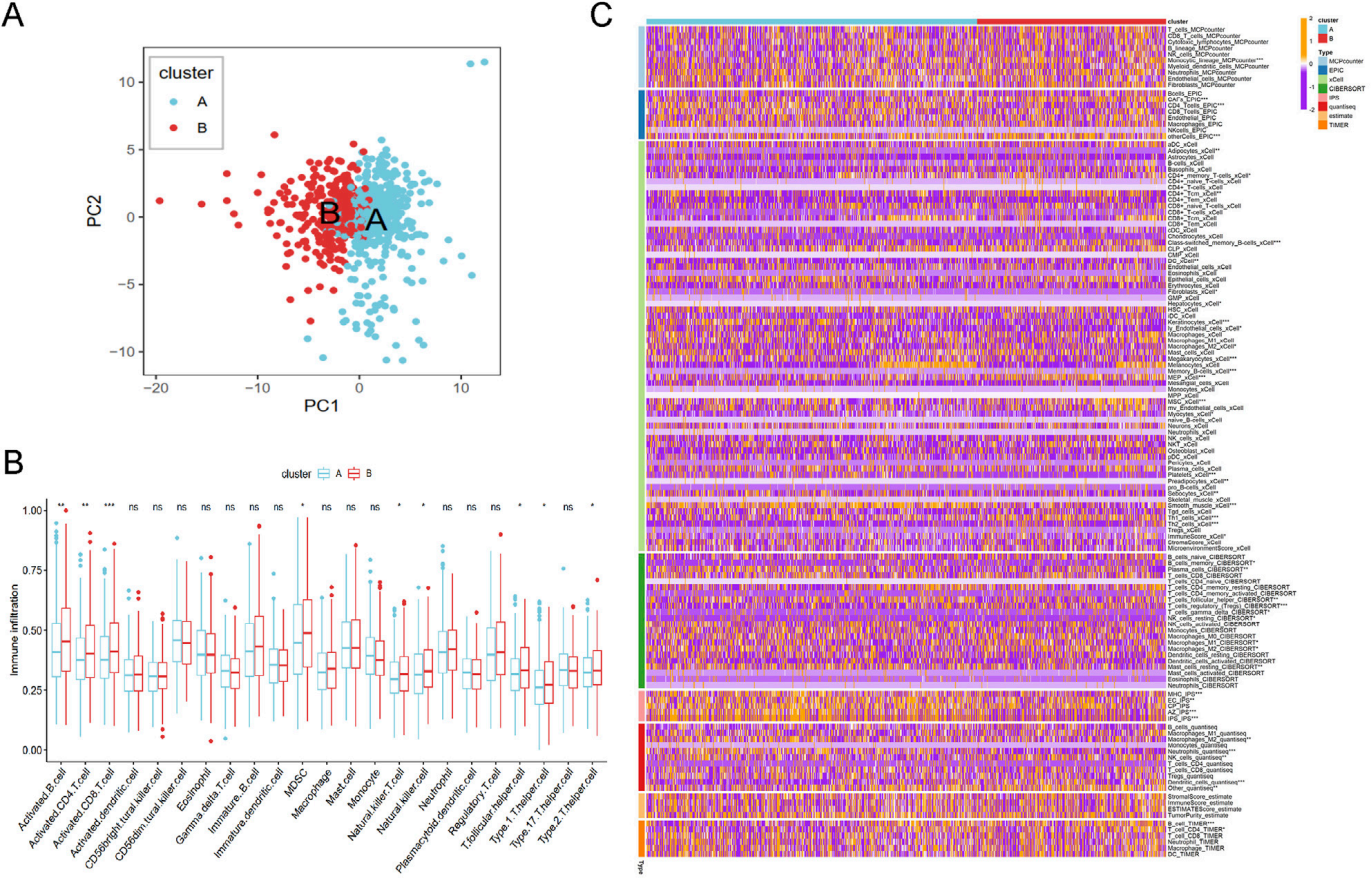
The immune analysis results between different cluster. **(A)** The PCA plot of cluster A and B. **(B)** The immune cells distribution based on ssGSEA algorithm. **(C)** Heatmap of tumor-related infiltrating immune cells based on TIMER, CIBERSORT, CIBERSORT-ABS, QUANTISEQ, MCPCOUNTER, XCELL, and EPIC methods.

Based on the principal component analysis (PCA) of prognostic genes, the samples in merged cohort could be divided into two cluster A and B (Figure 5A), aligning with the results from Figure 3B. Immune cell analysis revealed that Cluster B exhibited higher levels of activated B cells, activated CD4^+^ T cells, activated CD8^+^ T cells, MDSCs, natural killer T cells, natural killer cells, T follicular helper cells, Type 1 helper cells, and Type 2 helper cells (Figure 5B), consistent with immune infiltration results from eight different methods (Figure 5C). These biological characteristics of function enrichment indcated that Cluster B was activated in cancer related pathways, while Cluster A was characterized by distinct metabolic states. Despite the activated immune cells in Cluster B, the presence of immunosuppressive cells was also notable.

### Construction of a prognosis signature based on integrative machine learning

**FIGURE 6:**
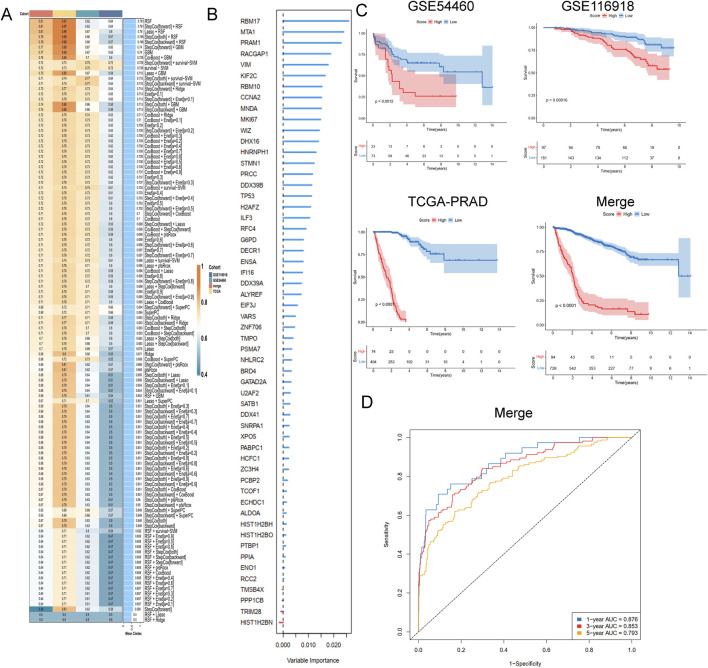
A consensus LRGRS was developed and validated via the machine learning-based combined procedure. **(A)** A total of 10 kinds of machine learning via a tenfold cross-validation framework and further calculated the C-index of each model across all validation datasets. **(B)** The barplot of hub genes based on forest trees methods. **(C)** Kaplan–Meier curves of OS according to the LRGRS in the GSE54460, GSE116918, TCGA-PRAD and combined cohorts, based on the log-rank test. **(D)** The ROC curves in combined cohorts.

**FIGURE 7:**
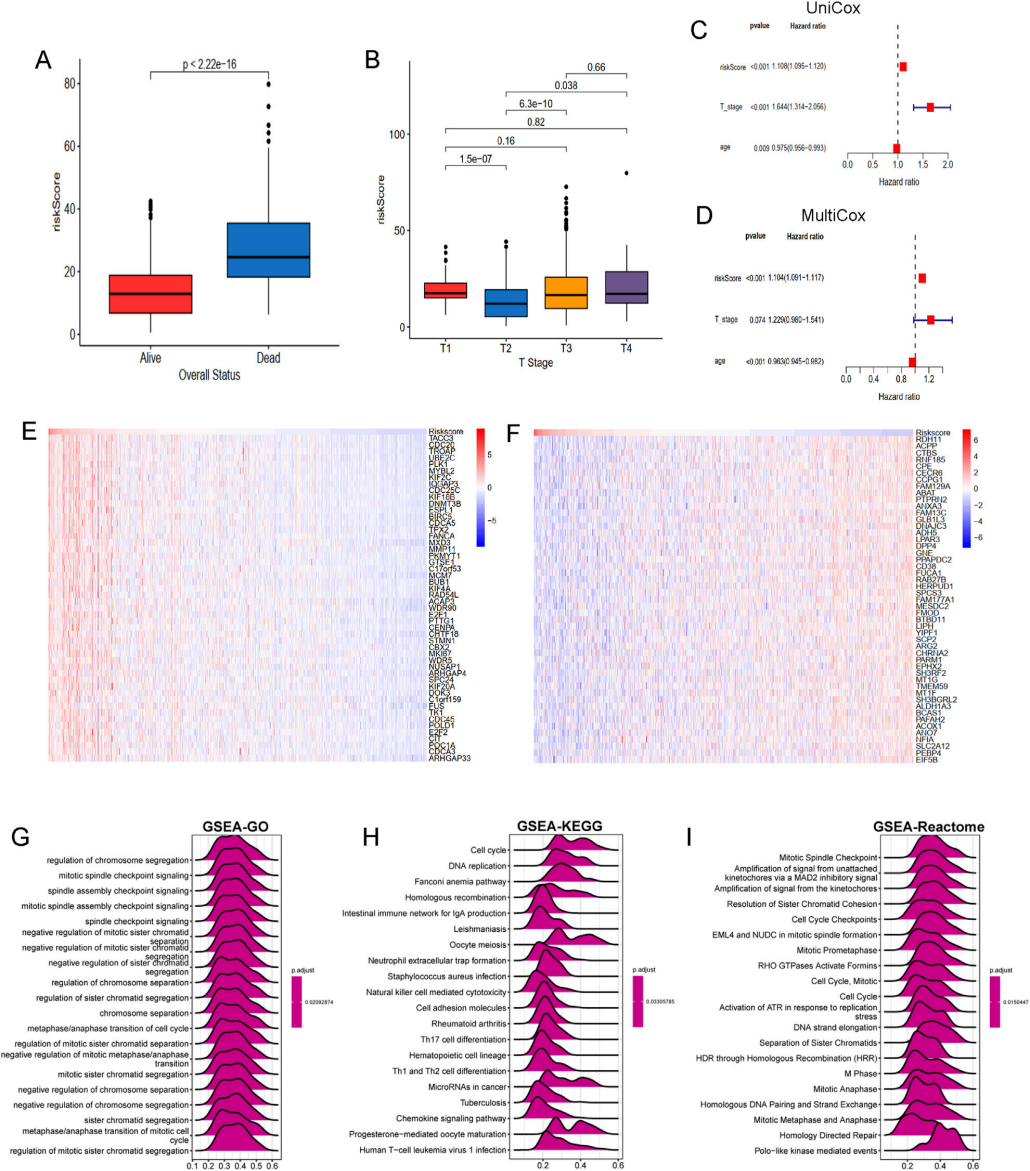
The association of LRGRS and clinical features, functional enrichments between different risk groups. **(A)** The boxplot of overall status and riskScore. **(B)** The boxplot of T stage and riskScore. The results of **(C)** univariate and **(D)** multivariate cox regression analysis. The mRNA expression profiles of positive genes **(E)** and negative genes **(F)** correlation with risk score. The GSEA results of GO **(G)**, KEGG **(H)** and Reactome **(I)** signal pathways.

To develop a consensus LRG signature (LRGS), ten machine-learning algorithms were employed to analyze 56 prognostic genes identified via univariate Cox regression. A total of 101 prediction models were applied to the merged dataset using tenfold cross-validation, and C-index values were calculated for all training and validation sets (Figure 6A). After extensive screening, the RSF model was identified as the most accurate and clinically relevant predictive model (Figure 6B). In the combined cohort, patients classified as high-risk had significantly poorer RFS compared to those in the low-risk category (p < 0.001, log-rank test). The GSE116918, GSE54460, and TCGA-PRAD datasets further confirmed that RFS was significantly improved in the low-risk group (p < 0.001, log-rank test; Figure 6C). ROC curve analysis showed that the LRGS achieved AUC values of 0.876, 0.853, and 0.793 for 1-, 3-, and 5-year intervals, respectively (Figure 6D). The correlation between the prognostic signature’s risk score and clinical characteristics revealed that higher death rates and higher T stages were associated with higher risk scores (Figures 7A,B). Univariate and multivariate Cox regression analyses on the prognostic risk scores (PRS) within the combined cohort identified LRGS as an independent prognostic factor for patients with PRAD, with a hazard ratio of 1.108 (95% CI: 1.095–1.120, p < 0.001) (Figures 7C,D).

### Molecular mechanisms underlying LRGS in bulk transcriptomics

To further elucidate the molecular mechanisms linking the LRGS with prognosis in PRAD, genes positively and negatively correlated with risk scores were identified. Several genes, such as TACC3, CDC20, TROAP, UBE2C, MYBL2 are positive associated with risk score (Figure 7E), while genes such as RDH11, ACPP, CTBS, RNF185, CPE are negative with risk score (Figure 7F). Functional enrichment analysis was performed using the GSEA method, revealing that the GO gene set was enriched in pathways related to chromosome segregation regulation, mitotic spindle checkpoint signaling, and spindle assembly checkpoint signaling (Figure 7G). KEGG pathway analysis indicated gene enrichment in pathways associated with the cell cycle (including cell cycle and DNA replication) and immune processes (such as neutrophil extracellular trap formation, NK cell-mediated cytotoxicity, and Th17, Th1, and Th2 cell differentiation) (Figure 7H). Reactome pathway enrichment analysis (Figure 7I) further confirmed these results, showing strong association with cancer-related biological processes and immune-related pathways.

### The correlation of immune microenvironment and immune characteristics with the LRGS

**FIGURE 8:**
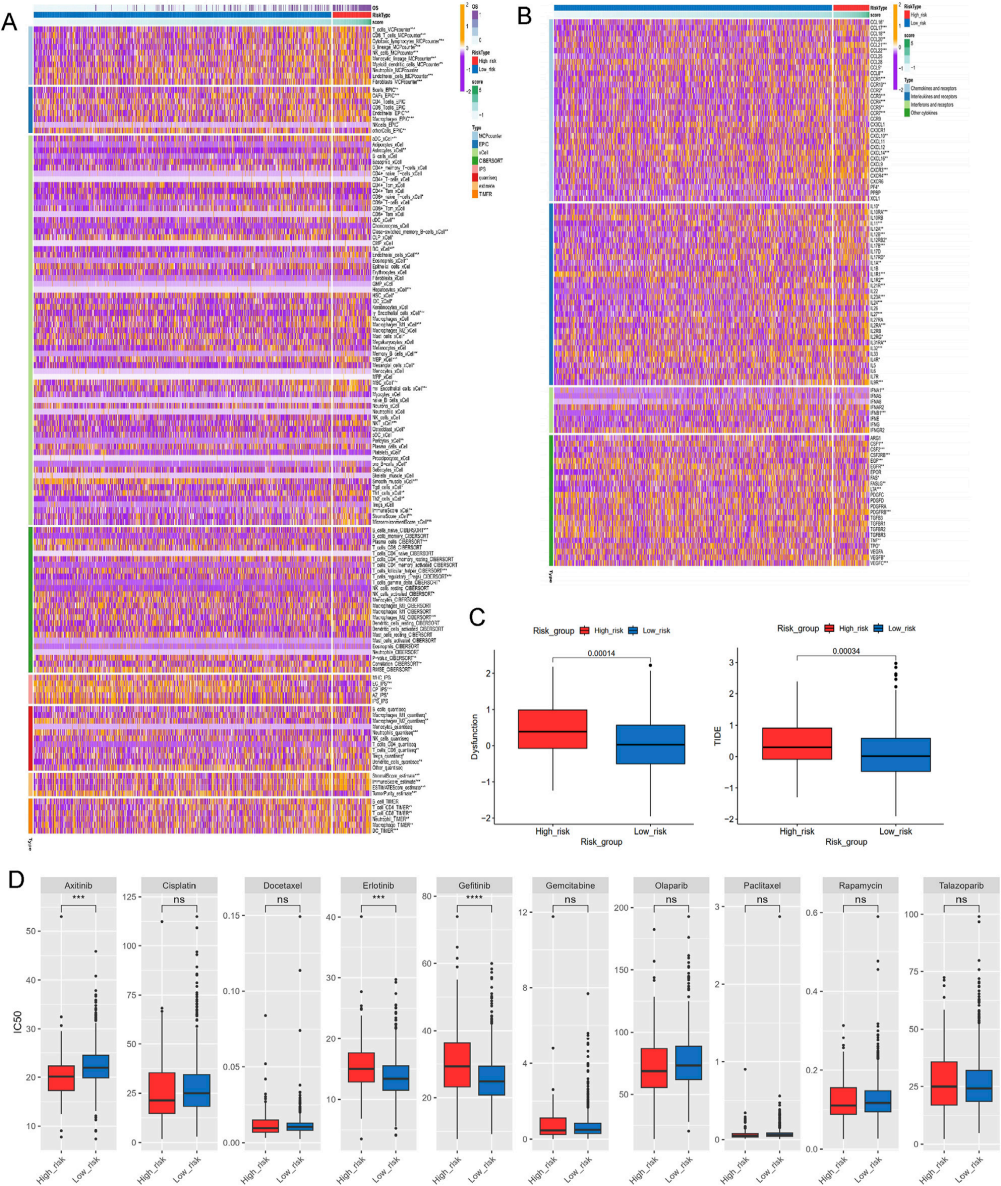
Investigations of immune profiling, immune score and drug sensitivity. **(A)** Heatmap of tumor-related infiltrating immune cells based on TIMER, CIBERSORT, CIBERSORT-ABS, QUANTISEQ, MCPCOUNTER, XCELL, and EPIC algorithms. **(B)** Heatmap of immune-related genes. **(C)** The immune function scores based on TIDE dataset. **(D)** Estimated IC50 of the indicated molecular-targeted drugs. ns > 0.05, p < 0.05, **p < 0.01, ***p < 0.001, ****p < 0.0001. ns, no significance.

A series of algorithms were used to investigate the TME across different risk score groups. The high-risk group exhibited reduced levels of T cells, CD8T cells, cytotoxic lymphocytes, NK cells, monocytes, and other immune cell types compared to the low-risk group (Figure 8A). Additionally, expression levels of chemokines (such as CCL5, CCL8, CCL16-18, CCL20-22), interleukins (such as IL10, IL11, IL12A-B, IL17, IL23A, IL24, IL27, IL31), interferons (such as IFNA1, IFNB1), and receptors were significantly different between the high- and low-risk groups (Figure 8B). TIDE and dysfunction scores were calculated using the TIDE dataset, indicating that the high-risk group exhibited elevated dysfunction and TIDE scores, suggesting immune effector cell exhaustion in high-risk samples (Figure 8C).

To address the malignant potential of PCa, this study explored various drug databases to identify therapeutic agents tailored to specific subtypes, focusing on the different risk score groups. The high-risk group demontrated resistance to targeted therapies such as erlotinib and gefitinib, but exhibited increased sensitivity to axitinib (Figure 8D). This highlights potential therapeutic strategies for PCa based on varying lactylation risk scores.

### The correlation of the top ten hub genes with single-cell characteristics

**FIGURE 9:**
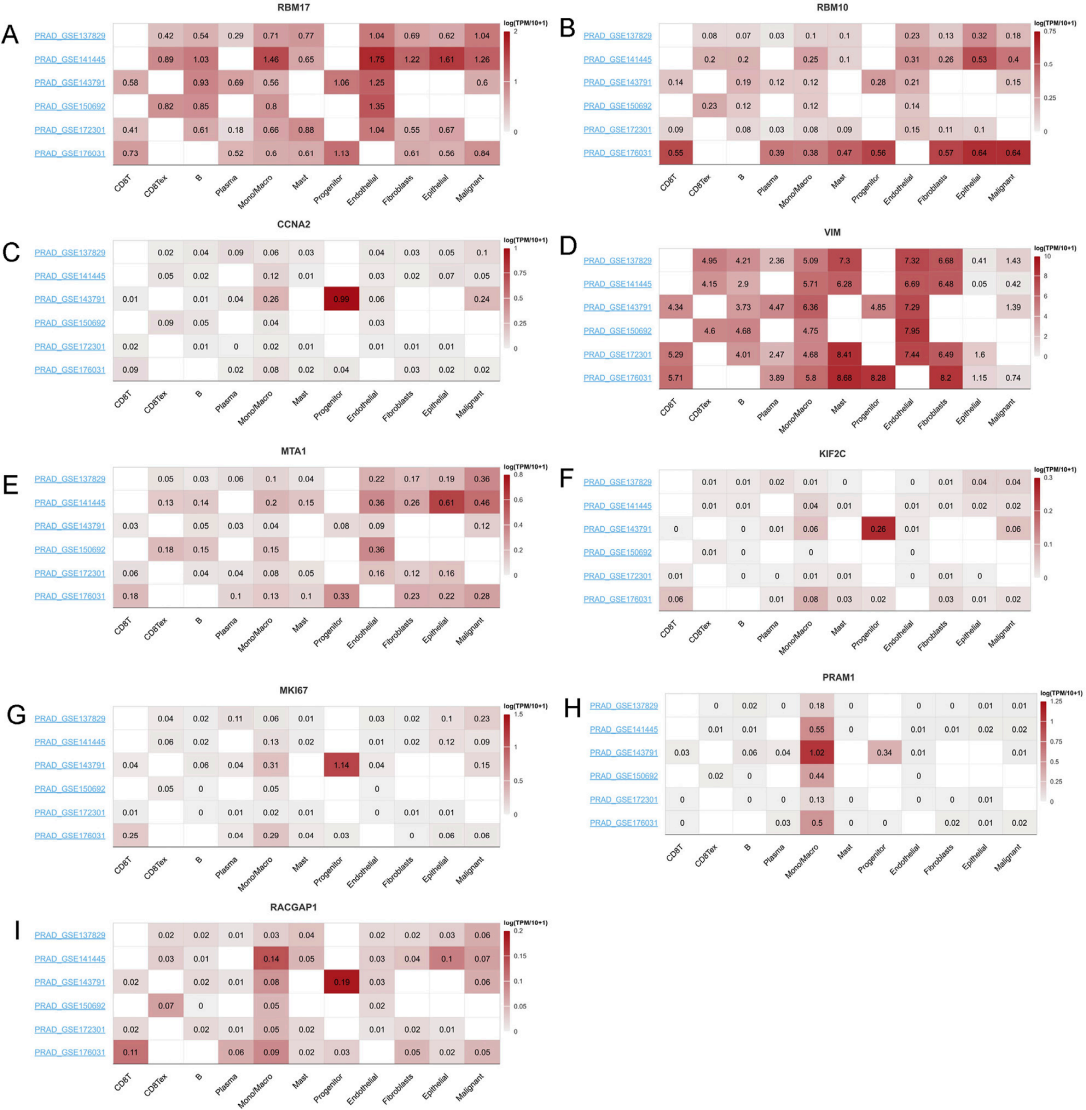
The mRNA expression profiles top ten genes in single cell sequencing levels based on TISCH2 dataset. The mRNA expression profiles of **(A)** RBM17, **(B)** RBM10, **(C)** CCNA2, **(D)** VIM, **(E)** MTA1, **(F)** KIF2C, **(G)** MKI67, **(H)** PRAM1 and **(I)** RACGAP1 in different PRAD datasets.

Given their cirtical roles in PCa, then we selected the top ten genes, RBM17, MTA1, PRAM1, RACGAP1, MKI67, MNDA, CCNA2,VIM,MNDA and RBM10 for further analysis. In TISCH2 datasets, MNDA information is not found. Then we only included other nine genes. The results showed that RMB17, RBM10, MTA1, VIM and RACGAP1 were enriched in endothelial, fibroblasts, epithelial and malignant (Figures 9A,B,D,E,I). As for CCNA2, KIF2C, MKI67 and PRAM1 were enriched in monocytes, macrophage and progenitor cells (Figures 9C,F–H). These findings aligned with immune cell infiltration patterns identified through various TME methodologies (Figure 8A).

To assess the prognostic relevance of LRGS, we performed Scissor analysis, an integrative method that links single-cell transcriptomic data with bulk-level phenotypes. By incorporating bulk RNA-seq expression profiles and corresponding survival information from the merged cohort, the Scissor algorithm classified single cells into three distinct groups: Scissor + cells, associated with poorer prognosis; Scissor− cells, linked to better survival outcomes; and background cells with no significant phenotype association (Supplementary Figure S1). Notably, macrophage/monocyte and fibroblast populations exhibited consistent associations across different Scissor analysis iterations, underscoring their potential roles in modulating prognostic phenotypes.

### Analysis of the multi-omics characteristics of the hub genes and validation of gene expression in PRAD

**FIGURE 10:**
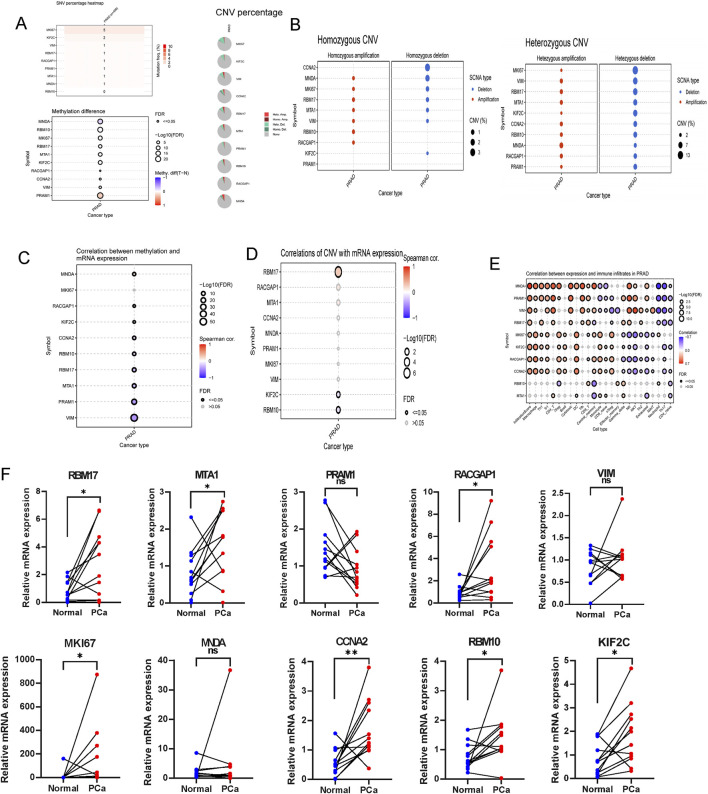
The association of gene mutations, CNV, methylation, immune infiltration and mRNA expression profiles in TCGA-PRAD, and validation in ten paired clinical samples. **(A)** The summary of SNV percentage, CNV percentage and methylation difference. **(B)** The homozygous and heterozygous CNV of TCGA-PRAD. The correlations between of top ten genes mRNA expression with methylation **(C)**, CNV **(D)** and immune infiltration **(E)**. **(F)** qRT-PCR analysis of hub genes expression in prostate cancer and paired adjacent normal tissues based on patient samples from Sir Run Run Shaw Hospital. ns > 0.05, p < 0.05, **p < 0.01, ***p < 0.001, ****p < 0.0001. ns, no significance.

Using the GSCA dataset, gene expressions levels of hub top genes and associated with SNV percentage, CNV percentage, methylation. The results demonstrated that MKI67 and KIF2C had 5%, 2% SNV percentage. PRAM1, MNDA and RBM10 had higher methylation levels (Figure 10A). The homozygous CNV showed that MNDA, MKI67, RBM17, MTA1, VIM, RBM10 and RACGAP1 had high homozygous amplication, CCNA2, MNDA, MKI67, RBM17, MTA1, VIM and KIF2C had high homozygous deletion. As for heterozygous CNV, these ten hub genes had high heterozygous CNV (Figure 10B). Subsequcently, the correlation between mRNA expression of ten hub genes and methylation, CNV levels were explored. These ten hub genes, expect MKI67, had significant difference with methylation (Figure 10C). As for the correlation with CNV levels, only RBM17, KIF2C and RBM10 had significant difference (Figure 10D). The analysis revealed that the majority of hub genes exhibited a positive correlation with macrophages, Th1, Tr1, CD4 T cells, iTreg, DC, Tfh cells, as well as CD4 naïve, Th17, and neutrophil cells (Figure 10E).

RT-qPCR was performed to assess the mRNA expression levels of the ten hub genes in clinical samples using RT-qPCR.The results indicated that the majority of signature genes (RBM17, MTA1, RACGAP1, MKI67, CCNA2, RBM10 and KIF2C) were expressed at higher levels in PCa tissues compared to adjacent normal samples (Figure 10F).

## Discussion

Recent estimates from GLOBOCAN 2024 indicate that PCa remains a leading cause of cancer incidence and mortality, with significant variations across different continents and among various ethnic groups (Bray et al., 2024; Gizzi et al., 2024). This disparity highlights the urgent need for a personalized approach to understanding, diagnosing, and treating PCa. A thorough understanding of its complexity is vital for developing individualized treatment strategies. This study advances the understanding of lactylation, a crucial PTM that influences gene expression and cellular metabolism in cancer cells (Li et al., 2024; Zhang Q. et al., 2024; Zhou et al., 2023).

Our single-cell RNA sequencing analysis reveals significant cellular heterogeneity within PCa, identifying distinct cell types exhibiting diverse lactylation profiles. This cellular diversity mirrors the broader genetic and metabolic heterogeneity of PCa, which is characterized by numerous genomic alterations and extensive metabolic reprogramming (Baca et al., 2013; Barbieri et al., 2012). The identification of LRGs as potential prognostic biomarkers marks a key step toward personalized medicine, enabling the stratification of patients based on LRG expression profiles.

Metabolic reprogramming, particularly the shift toward aerobic glycolysis, is a hallmark of PCa cells. This shift, characteristic of the Warburg effect, results in lactate accumulation, which, through lactylation, can modulate gene expression and promote tumor growt (Zhang X. et al., 2024; Pan et al., 2024). These insights into the immunomodulatory effects of lactate provide a rationale for targeting lactylation as part of immunotherapeutic strategies for PCa.

The TME plays a pivotal role in cancer progression, and our study demonstrates that lactate can profoundly influence the immune landscape within this environment. Lactate-induced acidification of the TME impairs T lymphocyte function and promotes the polarization of TAMs toward the protumorigenic M2 phenotype (Loeb et al., 2014).

These insights into the immunomodulatory effects of lactate provide a rationale for targeting lactylation as part of immunotherapeutic strategies for PCa.

The prognostic biomarker developed in this study, based on LRGs, offers a novel method for predicting treatment response and disease-free survival in patients with PCa. The use of machine learning algorithms to identify this biomarker emphasizes the potential of integrating computational methods with biological data to propel personalized medicine (Zeng et al., 2024). The identification of key genes such as TACC3, CDC20, and UBE2C, which are positively correlated with risk scores, lays the groundwork for further investigation into the molecular mechanisms underlying lactylation’s role in PCa.

The correlation between lactylation status and the immune microenvironment indicates that high-risk groups may exhibit a reduced presence of immune cells, potentially contributing to treatment resistance. This observation, coupled with the finding that high-risk groups may be more resistant to certain targeted therapies but show sensitivity to others, such as axitinib, underscores the critical need for personalized treatment strategies (Barbieri et al., 2012; Bray et al., 2018).

In summary, this study provides an in-depth analysis of lactylation in PCa, emphasizing its roles in tumor biology, immune evasion, and prognosis. The identification of LRGs as a prognostic biomarker, along with insights into their molecular mechanisms, forms the basis for the development of novel therapeutic strategies (Pan et al., 2024). Future research should aim to validate these findings in larger patient cohorts and explore lactylation as a potential therapeutic target. Personalized treatment strategies based on lactylation profiles have the potential to revolutionize PCa management, ultimately improving patient outcomes.

## Conclusion

This study comprehensively analyzed LRGs in PCa through single-cell and bulk RNA sequencing data. This multifaceted approach enabled the identification and characterization of LRG expression patterns across various cell types within the TME. The prognostic significance of these genes was confirmed by classifying clinical samples into two distinct subtypes, each associated with different tumor-related pathways, metabolic processes, and immune profiles. A machine learning-based prognostic signature model was developed, demonstrating high accuracy and offering new insights into personalized treatment strategies for PCa. This innovative model enhances our understanding of the molecular mechanisms driving PCa and provides a valuable tool for predicting treatment response and disease outcomes, ultimately facilitating more effective clinical management.

## Data Availability

The raw data supporting the conclusions of this article will be made available by the authors, without undue reservation.

## Supplementary material

SUPPLEMNTARY FIGURE S1 Correlation of LRGS with survival phenotype at single cell scale identified by the Scissor methods. **(A)** tSNE plots showcasing the survival phenotypes. **(B)** Balloonplot shows the proportation of cells based on cox methods. **(C)** tSNE plot shows the LRGS high and low phenotypes. **(D)** Balloonplot shows the proportation of cells based on logistic methods. **(E)** The proportation of cells among survival and LRGS phenotypes.
