# Supplementary figures and images for: Integrative analysis of lactylation related genes in prostate cancer: unveiling heterogeneity through single-cell RNA-seq, bulk RNA-seq and machine learning
Source: Front Pharmacol. 2025 Aug 4;16:1634985. doi: 10.3389/fphar.2025.1634985 (PMC12358452; doi:10.3389/fphar.2025.1634985)

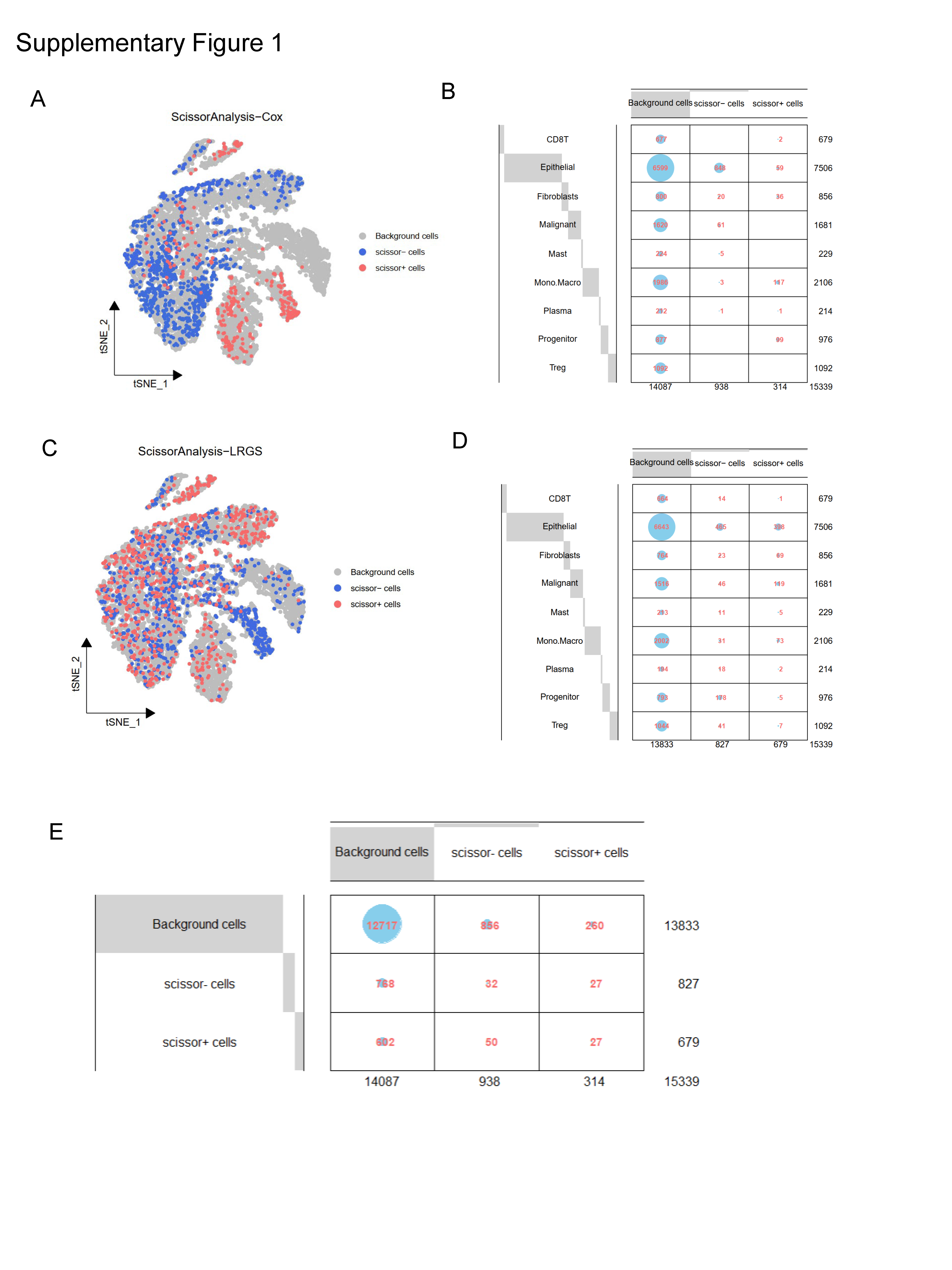

Supplement: Supplementary file 1 [file Supplementaryfile1.tiff]
